# QuickStats

**Published:** 2014-05-30

**Authors:** FROM THE NATIONAL CENTER FOR HEALTH STATISTICS

**Figure:**
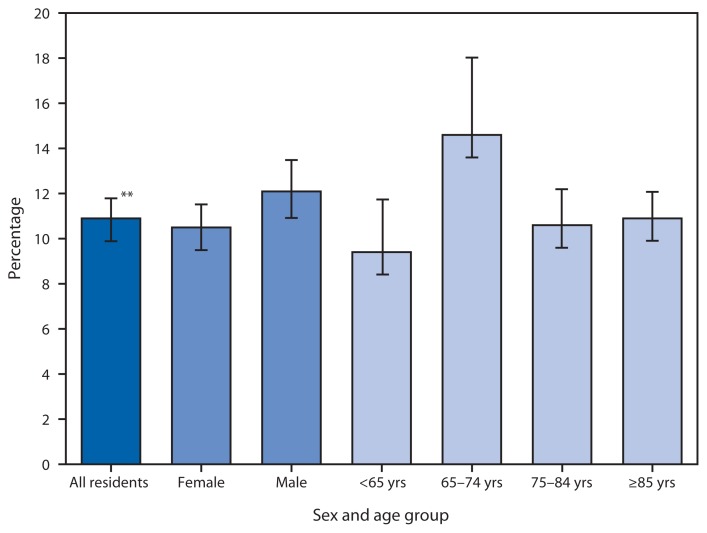
Prevalence of Stroke* Among Residential Care Residents,^†^ by Sex^§^ and Age Group^¶^ — National Survey of Residential Care Facilities, United States, 2010 * Respondents, who typically were residential care community directors, were asked, “As far as you know, has a doctor or other health professional ever diagnosed this resident with a stroke?” ^†^ Residential care residents refer to persons living in assisted living and similar places (e.g., personal care homes and adult care homes, board and care homes, and adult foster care) on any given day in 2010. Residents in nursing homes were excluded. Those with missing data for stroke (0.6%) were excluded. ^§^ Differences between female and male residents significant at p<0.10. ^¶^ Differences between residents aged 65–74 years and residents in the other three age groups were statistically significant at p<0.05. ** 95% confidence interval.

In 2010, approximately 11.0% of residential care residents had been diagnosed with a stroke. About 12.0% of male residents and 10.5% of female residents had been diagnosed with a stroke. Residents aged 65–74 years had the highest prevalence of stroke (14.6%) compared with the other age groups.

**Source:** National Survey of Residential Care Facilities, 2010. Available at http://www.cdc.gov/nchs/nsrcf.htm.

**Reported by:** Christine Caffrey, PhD, gwo9@cdc.gov, 301-458-4137; Manisha Sengupta, PhD.

